# Targeting splenic hematopoietic stem cells in cardiovascular disease

**DOI:** 10.18632/oncotarget.4918

**Published:** 2015-07-21

**Authors:** Matthias Nahrendorf, Partha Dutta

**Affiliations:** Center for Systems Biology, Massachusetts General Hospital and Harvard Medical School, Boston, MA, USA

**Keywords:** macrophage, hematopoietic stem cells, atherosclerosis

Inflammation plays a crucial role in cardiovascular disease. For example, inflammatory monocytes, after being recruited to atherosclerotic plaques, secrete proteinases that erode the fibrous cap. This may lead to plaque rupture and subsequent myocardial infarction (MI). Patients with acute myocardial infarction have elevated levels of blood leukocytes [1]. Peripheral blood leukocyte count after MI correlates with in-hospital mortality, underscoring role of inflammatory cells in post-MI myocardial remodeling.

The spleen provides steady flow of leukocytes to the infarct and atherosclerotic plaques. Since monocyte turnover at the sites of inflammation is very high [2], hematopoietic stem and progenitor cells (HSPC) differentiate into myeloid cells to keep up with the increased demand. MI drives splenic HSPC into the cell cycle [3] and induces their lineage differentiation. Subsequent imaging studies [4, 5] suggested splenocyte proliferation after acute myocardial infarction in patients. Although splenic myelopoiesis exaggerates inflammation in cardiovascular disease, the components of splenic hematopoietic niche are not well understood.

Since macrophages play a major role in bone marrow HSC maintenance, we investigated if macrophages regulate hematopoiesis in the spleen [6]. Toward this end, we developed lipidoid nanoparticles containing siRNA against CD115, the receptor for MCS-F, which is involved in macrophage differentiation, proliferation and survival. CD115 knockdown significantly reduced splenic macrophage numbers in mice injected with the Toll like receptor ligand lipopolysaccharide. Concomitantly, splenic HSC, HSPC and granulocyte macrophage progenitor numbers were diminished after knocking down CD115. Levels of VCAM-1, an HSC retention factor, were reduced in the spleen; however, HSC proliferation and apoptosis were unaltered, indicating impaired retention of splenic HSC after CD115 knockdown. Reduced splenic retention was in line with elevated HSPC levels in the blood after siCD115 treatment. Additionally, we found similar diminished splenic HSC numbers after depletion of splenic macrophages in CD169-iDTR mice with diphtheria toxin, bolstering the hypothesis that macrophages maintain splenic HSC. CD115 knockdown curtailed myeloid cell supply to the infarct in mice on day 4 after coronary ligation. Furthermore, the treatment also abated inflammation in atherosclerotic lesions in ApoE−/− mice fed with high fat diet. This reduction of inflammation was likely a consequence of mitigated extramedullary myelopoiesis due to impaired HSC maintenance after CD115 knockdown. In agreement with reduced inflammation, the treatment reduced plaque size and necrotic core area, and thickened fibrous cap. These are features of stable atherosclerotic plaques.

Since VCAM-1 was the only HSC retention factor that was downregulated in splenocytes after CD115 knockdown, we investigated which hematopoietic cells express VCAM-1. Out of all hematopoietic cells tested, only macrophages expressed VCMA-1 at high levels. An adoptive transfer experiment revealed that splenic HSPC reside in close proximity with VCAM-1^+^ macrophages. In fact, more than 90% of HSPC reside within 10 μm distance from this macrophage subset. These data indicate that VCAM-1^+^ macrophages maintain splenic HSC. To investigate this further, we formulated siRNA against VCAM-1 in lipidoid nanoparticles which enrich in macrophages but not endothelial cells. VCAM-1 knockdown in macrophages significantly reduced myelopoiesis and HSC numbers in the spleen. Of note, the treatment did not change HSC numbers in the bone marrow, indicating involvement of other cell types and redundant mechanisms of retention for bone marrow HSC maintenance. Further, macrophage-specific VCAM-1 knockdown reduced inflammation in atherosclerosis and improved characteristics of stable atherosclerotic plaques.

In summary, the study suggests that VCAM-1^+^ macrophages are important components of splenic HSC niche. In absence of VCAM-1 or splenic macrophages, HSC depart from the spleen, resulting in decreased myelopoiesis after MI and in mice with atherosclerosis. We previously showed that MI-induced splenic HSPC proliferation is stem cell factor-dependent [3]. Endothelial cells are major source of stem cell factor, which maintain HSC in the bone marrow [7]. However, it is not known if SCF secreted by splenic endothelial cells after MI triggers myelopoiesis. The bone marrow HSC niche is a complex microenvironment and orchestrated by different cells such as nestin^+^ mesenchymal stem cell, endothelial cells and regulatory T cells. It remains to be investigated which splenic cells besides macrophages maintain HSC.
